# Overview and Insights into Carbapenem Allergy

**DOI:** 10.3390/pharmacy7030110

**Published:** 2019-08-08

**Authors:** Yuman Lee, Nicole Bradley

**Affiliations:** College of Pharmacy and Health Sciences, St. John’s University, Queens, NY 11439, USA

**Keywords:** carbapenem, allergy, cross-sensitivity, imipenem/cilastatin, meropenem, ertapenem, doripenem

## Abstract

Understanding antibiotic allergies and the risk of cross-sensitivity between and within antibiotic classes can have a substantial impact on patient care. The purpose of this review article is to provide insight into carbapenem allergies, describing the overall incidence, risk factors, and in-class cross-sensitivity. A PubMed search was conducted using the following search terms: carbapenem, allergy, cross-sensitivity, incidence, imipenem/cilastatin, meropenem, ertapenem, and doripenem. Article bibliographies and relevant drug monographs were also reviewed. The overall reported incidence of carbapenem allergy is 0.3%–3.7%. Risk of cross-sensitivity between penicillins and carbapenems is less than 1% in patients with a positive penicillin skin test. Data on cross-sensitivity between cephalosporins and carbapenems are limited; however, the risk appears to also be low. No clinical studies have described cross-sensitivity between the carbapenem agents thus far. The limited data available from case reports demonstrates a lack of cross-sensitivity between the individual carbapenems, suggesting that an alternative carbapenem may cautiously be used in patients with a reported carbapenem allergy.

## 1. Introduction

Carbapenem antibiotics are members of the beta-lactam class that are considered crucial to the management of various infectious diseases. Of the beta-lactams, carbapenems possess the broadest spectrum of activity, serving as highly-potent agents against both Gram-positive and Gram-negative bacteria, including anaerobic bacteria. Structurally, carbapenems are defined as the 4:5 fused beta-lactam ring of penicillins with a double bond between C-2 and C-3 and the substitution of a carbon atom for sulfur atom at C-1. This replacement of carbon for sulfur in the beta lactam ring is what differentiates carbapenems from penicillins [1]. The change allows for the broader spectrum of activity seen with carbapenems compared to penicillins and cephalosporins.

Thienamycin was the first carbapenem antibiotic to be discovered in 1976. Its clinical use was limited by the compound’s instability in water, but its discovery would later serve as the model compound for all carbapenem agents [2]. In 1985, imipenem/cilastatin received FDA approval, making it the first carbapenem agent available in the United States (U.S.) [3]. Today, there are four carbapenem agents approved for use in the U.S.: Imipenem, meropenem, ertapenem, and doripenem. In addition, two carbapenem/beta-lactamase inhibitor combinations, meropenem/vaborbactam and imipenem/cilastatin/relebactam, are also available on the U.S. market.

The purpose of this review is to provide insight into carbapenem allergies, describing the overall incidence, risk factors, and in-class cross-sensitivity.

A literature search of PubMed was conducted using the following search terms: carbapenem, allergy, cross-sensitivity, incidence, imipenem/cilastatin, meropenem, ertapenem, and doripenem. Article bibliographies and relevant drug monographs were also reviewed. Relevant English-language studies published before June 2019 were considered for inclusion.

## 2. Indications

Carbapenem antibiotics are FDA approved for use in a wide range of infections including respiratory tract infections, genitourinary tract infections, intra-abdominal infections, bacteremia, osteomyelitis, and skin and skin structure infections [3,4,5,6]. They are generally well tolerated, with the most common adverse reactions among the class being headache, nausea, diarrhea, vomiting, and rash. Additionally, the carbapenems carry warnings for seizures and other CNS adverse reactions [3,4,5,6].

Despite their numerous indications, it is important to recognize the role and utility of carbapenems in clinical practice. Because the carbapenems have activity against resistant Gram-negative bacteria, specifically extended-spectrum-beta-lactamase (ESBL) producing organisms, they have become a protected class of antibiotics and are often used as “last-line” agents, when use of no other beta-lactam agent would be acceptable for treatment.

Generally, carbapenem use is limited to patients who have infections with multidrug resistant organisms (MDROs) or risk factors for infection with MDROs [2]. However, with rates of Gram-negative resistance continuing to rise, carbapenem use is becoming more widespread in routine clinical practice.

Given the important role of carbapenems in clinical practice, understanding carbapenem allergy is essential for providers.

## 3. Incidence of Allergy

Generally, carbapenems are well tolerated with a low risk for allergic reactions. The reported incidence of rash, pruritus, and urticaria was 0.3%–3.7% in post-marketing studies of imipenem, meropenem, ertapenem and doripenem [7,8,9,10,11,12]. No patients from these studies experienced anaphylaxis, however patients with a prior history of anaphylaxis to any beta-lactam were excluded from some of these studies [8,9,10].

Shortly after its approval, the safety profile of imipenem/cilastatin in worldwide clinical experience was evaluated among 3470 patients. The study reports a 3% incidence of rash, pruritus, and urticaria, but notes that less than 50% of these dermatologic reactions were felt to be probably or definitely drug related. No patient developed life-threatening consequences from these reactions. Of note, some of the patients who developed a rash during imipenem/cilastatin therapy had a history of rashes when treated with other beta-lactams. None of the patients included had a prior history of anaphylactic reactions to other beta-lactams [8]. In the larger clinical trial of over 9000 patients treated with imipenem/cilastatin, zero patients had an anaphylactic reaction. Again, patients with a prior history of anaphylactic reactions to other beta-lactams were excluded [8,9].

In 2007, the safety profile of meropenem was evaluated in over 6000 patients who received treatment with meropenem. The evaluation included the safety analysis from clinical trial data as well as real world data with serious bacterial infections. Patients with a prior history of hypersensitivity to any beta-lactam were excluded. Rash occurred in only 1.4% of cases and pruritis in 0.3% of cases. Meropenem related anaphylaxis was not reported in any of the cases [10].

The safety of ertapenem was evaluated in 240 healthy volunteers and 2046 patients enrolled in Phase II and III clinical trials. In one small Phase I trial of 41 healthy volunteers, one person discontinued ertapenem due to urticaria, folliculitis, and gastrointestinal reflux. In larger Phase II and III clinical trials, the incidence of ertapenem related pruritus and rash was 0.5%–1.2% and 1.1%–1.6%, respectively. No cases of anaphylaxis were reported. The authors did not note if patients with known hypersensitivity to beta-lactams were included in this evaluation [11].

Lastly, the safety of doripenem was evaluated in seven clinical trials including over 1800 patients who received any dose or partial dose of meropenem. Hypersensitivity to doripenem was reported in 0.7% of patients. Pruritus and rash occurred in 1.8% and 3.7% of patients. Again, no cases of anaphylaxis were reported, however there is no indication of whether patients with known hypersensitivity to beta-lactams were included in this evaluation [12].

## 4. Risk Factors

Given the low incidence of carbapenem allergies reported in post-marketing studies, risk factors that predispose patients to a carbapenem allergy have not been described in the literature. However, due to the structural similarity of a beta-lactam ring that is common to all beta-lactam antibiotics, risk factors that have been described with penicillin and cephalosporin allergies could potentially be considered for carbapenem allergies. These risk factors include a history of beta-lactam allergy, female gender, increasing age, previous antibiotic exposures and adverse reactions, as well as genetic predispositions [13,14,15,16,17,18,19,20].

The potential for a carbapenem allergy in patients with beta-lactam allergies theoretically correlates to the incidence of cross-sensitivity between penicillins or cephalosporins and carbapenems. Historically, the cross-sensitivity between penicillins and carbapenems were reported to be as high as 47.4% [21]. However, this relatively high rate of cross-sensitivity was concluded from a non-validated method of imipenem skin testing and patients were not challenged with imipenem to confirm a true allergy. Since then, further studies with different patient populations and methodologies have reported a much lower cross-sensitivity rate between penicillins and carbapenems [21,22,23]

In a systematic review published in 2014, the incidence of carbapenem hypersensitivity, of any type, among 838 patients with previous IgE-mediated penicillin reactions was 4.3% (95% CI, 3.1%–5.9%) [24]. Of the subset of patients with a positive penicillin skin test, only 0.3% (1/295) of patients had a hypersensitivity reaction to a carbapenem (95% CI, 0.06%–1.9%) [24]. A prospective study published after the meta-analysis also reported a low cross-sensitivity rate between penicillins and carbapenems [25]. This study included 212 patients with immediate reactions to penicillins and positive penicillin skin test results. The patients all underwent skin testing with carbapenems (imipenem, meropenem, and ertapenem) and all of the patients had a negative skin test result. Of the 211 patients who consented to a graded challenge, all of the patients tolerated carbapenems [25].

There is a paucity of data on the cross-sensitivity between cephalosporins and carbapenems. In the same systematic review aforementioned, the incidence of cross-sensitivity in patients with a previous proven, possible, or suspected IgE-mediated cephalosporin reaction to carbapenems was 25% (3/12) [24]. The hypersensitivity reactions to carbapenems included two non-IgE mediated reactions and one IgE-mediated reaction. In the largest prospective study available, which included 98 patients with confirmed IgE-mediated hypersensitivities to cephalosporins, only one patient had a positive skin test to both cephalosporins and carbapenems (imipenem and meropenem) [26]. A graded challenge was performed in the 97 patients who had negative carbapenem skin test results. Only one patient did not tolerate the graded challenge and developed a mild urticarial reaction to imipenem 30 min after the full dose [26].

Risk factors associated with carbapenem allergies have not been clearly identified due to the infrequency of carbapenem allergies. It may be reasonable to extrapolate these risk factors from those patients with penicillin and cephalosporin allergies. From this data, the risks of carbapenem allergies in patients who are allergic to penicillins and cephalosporins appear to be very low. The cross-sensitivity rates between cephalosporins and carbapenems may be slightly higher, however minimal data were available.

## 5. In-Class Cross-Sensitivity

Based on the similar core structures of the available carbapenems, a high rate of in-class cross-sensitivity may be expected, though no clinical studies have described cross-sensitivity between the carbapenems thus far. In animal models, anti-meropenem antibodies raised in rabbits and guinea pigs demonstrated a weak cross-sensitivity to imipenem [27]. Despite this finding, a few case reports have demonstrated the possible tolerability of an alternative carbapenem agent in the setting of documented allergy [28,29,30,31,32]. A summary of these reports can be found in Table 1.

In 2000, Chen and colleagues described a patient initially treated with topical imipenem/cilastatin for an open surgical wound after the repair of a distal right tibia and fibula fracture. After several days of treatment, she developed a generalized rash and itching which was resolved upon discontinuation of imipenem/cilastatin. Eight weeks later, the patient was given intravenous imipenem/cilastatin during a procedure and developed anaphylactic shock within 10 min of receiving the imipenem/cilastatin. Upon skin testing, the patient had a reaction to imipenem alone and no reaction to cilastatin. Specific IgE antibodies to imipenem were also found. Skin testing for 12 different antibiotic solutions, including meropenem, were negative [28].

In 2014, Bauer and colleagues reported a case of a patient who tolerated a course of meropenem after developing an allergic reaction to imipenem/cilastatin. The patient was initiated on imipenem/cilastatin for septic shock secondary to a leaking anastomosis and developed a large, erythematous, maculopapular rash within 48 h of initiation. Imipenem was subsequently discontinued, and the skin eruptions improved over the course of 7–10 days. Ten weeks later, the patient again required carbapenem therapy. She received a single challenge dose of meropenem 125 milligrams intravenously over 15 min and was observed by a nurse for an additional 15 min after the dose was administered. The meropenem dose was doubled and the patient was observed every 30 min until a meropenem dose of 1 g was achieved. The patient went on to complete a 14-day course of meropenem with no skin eruptions or change in hemodynamic state. The authors concluded that this case represented either a lack of cross-sensitivity between meropenem and imipenem or an allergy to cilastatin [29]. Of note, no other cases of confirmed or possible allergy cilastatin alone have been reported thus far.

Lakhal and colleagues reported a case of safe use of meropenem in a patient with a possible delayed reaction to imipenem. Their patient received an 8-day course of imipenem/cilastatin for the treatment of ventilator-associated pneumonia and developed an erythematous macular morbilliform rash and increased eosinophil count. Approximately 1 week later, the patient had recurrent pneumonia and was treated with a 14-day course of meropenem. No reaction occurred. Skin prick and intradermal testing completed 6 weeks later was negative at maximum concentrations of imipenem/cilastatin. Intradermal testing was positive when using high, possibly irritating, imipenem/cilastatin doses. Skin prick and intradermal testing was negative for meropenem and amoxicillin. The authors suggest that if carbapenem therapy is necessary, meropenem may be cautiously administered in patients with reported imipenem/cilastatin allergy [30].

Similarly, Noguerado-Mellado and colleagues described two cases of safe administration of an alternative carbapenem in patients with confirmed delayed onset imipenem and meropenem allergy. In case 1, the patient developed a delayed onset rash to imipenem/cilastatin but demonstrated tolerance to meropenem and other beta-lactams. In case 2, the patient developed a suspected type IV hypersensitivity to meropenem and showed tolerance to imipenem/cilastatin and other beta-lactams. The authors suggest that because of the lack of cross-sensitivity between imipenem and meropenem, the side chains of individual carbapenems likely play an important role in the development of delayed hypersensitivity reactions [31].

Most recently, Gil-Serrano and colleagues reported a case of anaphylactic shock to meropenem with tolerance to ertapenem. The patient was initiated on meropenem for septic shock and immediately developed itching, generalized erythema, and went into anaphylactic shock. Meropenem was discontinued and the patient’s infection was treated with ciprofloxacin. Initial antibiotic skin testing was performed 6 days after the anaphylaxis occurred. At that time, skin prick and intradermal testing with meropenem were negative. The patient was retested 4 weeks later, and both the skin prick and intradermal testing with meropenem were positive. Skin testing with imipenem/cilastatin and ertapenem were both negative. Later, the patient required carbapenem therapy and tolerated a 21-day course of ertapenem after a controlled challenge with ertapenem was performed. This case again shows a lack of cross-sensitivity between meropenem and other carbapenems, suggesting that an alternative carbapenem may be cautiously used in patients with a reported meropenem allergy [32].

## 6. Conclusions

Carbapenem antibiotics are generally well tolerated and associated with a low incidence of allergic reaction. Though no specific risk factors for allergy to carbapenems have been defined in the literature, potential cross-sensitivity in patients who have true penicillin or cephalosporin allergies may be a consideration. However, based on the data available, the risk of carbapenem allergy in patients allergic to penicillins or cephalosporins appear to be very low.

Cross-sensitivity between the individual carbapenem agents has not been well described in the literature. The majority of the evidence available is derived from case reports, which have demonstrated tolerability of an alternative carbapenem agent, even in cases of IgE mediated allergy. Of the six published cases, four involved patients who experienced imipenem/cilastatin allergic reactions, but were able to tolerate meropenem therapy or had negative skin tests for meropenem. The remaining two cases involved allergic reactions to meropenem. One of these patients was not re-challenged with a carbapenem, but had negative skin test results for imipenem/cilastatin. The other patient had negative skin tests for imipenem/cilastatin and ertapenem, and was successfully treated with ertapenem. These findings suggest that the side-chains of individual carbapenems may play an important role in the development of allergy. Based on the limited data available, an alternative carbapenem agent may be cautiously used in patients with reported carbapenem allergy if no other options for treatment are available.

## Figures and Tables

**Table 1:** Summary of case reports on carbapenem in-class cross-sensitivity.

Author/Year of Publication	Study Design	Characteristics	Findings
Chen et al., 2000 [28]	Case Report	● Treated with topical imipenem/cilastatin for open surgical wound ■ Developed generalized rash and itching several days into treatment ■ Resolved upon discontinuation ● Re-challenged with imipenem/cilastatin 8 weeks later ■ Developed anaphylactic shock within 10 min of re-challenge	Skin testing showed reaction to imipenem and no reaction to cilastatin IgE antibodies to imipenem found Negative skin test results for meropenem
Bauer et al., 2004 [29]	Case Report	● Treated with imipenem/cilastatin for septic shock ■ Developed large, erythematous, maculopapular rash within 48 h ■ Resolved within 7–10 days upon discontinuation ● Challenged with meropenem 10 weeks later ■ Dose gradually titrated up ■ Tolerated 14 days of meropenem therapy with no reaction	● No skin test results reported ● Safe administration of meropenem in patient with reaction to imipenem/cilastatin ■ Gradual dose increase of meropenem
Lakhal et al., 2007 [30]	Case Report	● Treated with imipenem/cilastatin for ventilator associated pneumonia ■ Developed erythematous macular morbilliform rash and increased eosinophil count after 5 days of treatment ● Challenged with meropenem 1 week later ■ Tolerated 14-day course with no reaction	● Prick and intradermal (ID) skin testing completed 6 weeks later: ■ Negative results at maximum imipenem/cilastatin doses ■ Positive ID test when high, possibly irritating, dose of imipenem/cilastatin used ■ Negative skin test results for meropenem and amoxicillin ● Safe administration of meropenem in patient with reaction to imipenem/cilastatin
Noguerado-Mellado et al., 2014 [31]	2 Case Reports	Case 1: ● Treated with imipenem/cilastatin ■ Developed delayed onset micropapular exanthema ● Challenged with meropenem 6 years later ■ Tolerated course with no reaction Case 2: ● Treated with meropenem ■ Developed generalized scaly erythematous rash with desquamation 3–4 days after initiation ■ Recovered in approximately 25 days ● Not challenged with another carbapenem	Case 1: Positive delayed reading ID skin test to imipenem Negative delayed reading ID skin test to cilastatin Negative delayed reading ID skin tests to meropenem, penicillin, amoxicillin, cefuroxime and ceftriaxone Safe administration of meropenem in patient with reaction to imipenem Case 2: Positive delayed reading ID skin test to meropenem Negative delayed reading ID skin tests to imipenem/cilastatin, amikacin, penicillin G, amoxicillin, cefuroxime, and ceftriaxone
Gil-Serrano et al., 2019 [32]	Case Report	● Treated with meropenem for septic shock ■ Developed immediate itching, generalized erythema and anaphylactic shock ■ Meropenem discontinued ● Challenged with ertapenem ■ Dose gradually titrated up ■ Tolerated 21-day course with no reaction	Initial skin prick and intradermal testing for meropenem negative Repeat skin prick and intradermal testing for meropenem positive 4 weeks later Skin testing for imipenem/cilastatin and ertapenem negative Safe administration of ertapenem in a patient reaction to meropenem
